# Return-to-Work Following Occupational Rehabilitation for Long COVID: Descriptive Cohort Study

**DOI:** 10.2196/39883

**Published:** 2022-09-14

**Authors:** Katelyn Brehon, Riikka Niemeläinen, Mark Hall, Geoff P Bostick, Cary A Brown, Marguerite Wieler, Douglas P Gross

**Affiliations:** 1 Department of Physical Therapy Faculty of Rehabilitation Medicine University of Alberta Edmonton, AB Canada; 2 Millard Health Treatment Centre Workers' Compensation Board of Alberta Edmonton, AB Canada; 3 Department of Occupational Therapy Faculty of Rehabilitation Medicine University of Alberta Edmonton, AB Canada

**Keywords:** compensation and redress, postacute COVID-19 syndrome, long COVID, COVID-19, rehabilitation, return-to-work, health outcome, occupational health, patient-reported outcome, anxiety disorder

## Abstract

**Background:**

Emerging evidence suggests that worldwide, between 30% and 50% of those who are infected with COVID-19 experience long COVID (LC) symptoms. These symptoms create challenges with return-to-work (RTW) in a high proportion of individuals with LC. To tailor rehabilitation programs to LC sequelae and help improve RTW outcomes, more research on LC rehabilitation program outcomes is needed.

**Objective:**

This study describes the characteristics and outcomes of workers who participated in an LC occupational rehabilitation program.

**Methods:**

A cohort study was conducted. Descriptive variables included demographic and occupational factors as well as patient-reported outcome measures (PROMs, ie, the Fatigue Severity Scale [FSS], the Post-COVID Functional Scale [PCFS], the 36-item Short Form Health Survey [SF-36], the Pain Disability Index [PDI], the pain Visual Analogue Scale [VAS], the 9-item Patient Health Questionnaire [PHQ-9], the 7-item Generalized Anxiety Disorder Questionnaire [GAD-7], and the Diagnostic and Statistical Manual for Mental Disorders Fifth Edition [DSM-5] posttraumatic stress disorder [PTSD] checklist [PCL-5]). The main outcome variable was the RTW status at discharge. Descriptive statistics were calculated. Logistic regression examined predictors of RTW.

**Results:**

The sample consisted of 81 workers. Most workers were female (n=52, 64%) and from health-related occupations (n=43, 53%). Only 43 (53%) individuals returned to work at program discharge, with 40 (93%) of these returning to modified duties. Although there were statistically significant improvements on the pain VAS (mean 11.1, SD 25.6, *t*_31_=2.5, *P*=.02), the PDI (mean 9.4, SD 12.5, *t*_32_=4.3, *P*<.001), the FSS (mean 3.9, SD 8.7, *t*_38_=2.8, *P*=.01), the SF-36 PCS (mean 4.8, SD 8.7, *t*_38_=–3.5, *P*=.001), the PHQ-9 (mean 3.7, SD 4.0, *t*_31_=5.2, *P*<.001), and the GAD-7 (mean 1.8, SD 4.4, *t*_22_=1.8, *P*=.03), there were no significant improvements in the PCFS, the overall mental component score (MCS) of the SF-36, or on the PCL-5. The availability of modified duties (odds ratio [OR] 3.38, 95% CI 1.26-9.10) and shorter time between infection and admission for rehabilitation (OR 0.99, 95% CI 0.99-1.00) predicted RTW even when controlling for age and gender.

**Conclusions:**

Workers undergoing LC rehabilitation reported significant but modest improvements on a variety of PROMs, but only 43 (53%) returned to work. Outcomes would likely improve with increased availability of modified duties and timelier rehabilitation. Additional research is needed, including larger observational cohorts as well as randomized controlled trials to evaluate the effectiveness of LC rehabilitation.

## Introduction

### Background

Emerging evidence indicates that worldwide, between 30% and 50% of those who contract COVID-19 experience long COVID (LC) symptoms (dependent on ethnicity, gender, and hospitalization status) [1]. LC is defined by the World Health Organization as postacute COVID-19 sequelae lasting at least 3 months postinfection that are not explained by any other diagnosis [2]. An international study (N=3762) analyzing the symptom makeup and severity, expected clinical course, impact on daily functioning, and return to baseline health of individuals experiencing LC found that the time to full recovery exceeded 35 weeks for most respondents (>91%) [3]. For some, the time to full recovery is much longer.

Individuals recovering from COVID-19 will increase demands for rehabilitation due to the prevalence and diversity of recognized LC sequelae [4-11]. Common LC symptoms, such as profound fatigue, breathlessness, cognitive impairment (brain fog), and muscle and joint pain, among other mental and physical health symptoms, create challenges with return-to-work (RTW) [11-13]. A systematic review (N=81 studies) found that between 29% and 47% of those employed prior to contracting COVID-19 were unable to RTW [14]. RTW with LC was found to be most limited when symptoms included fatigue and cognitive impairment [14-17]. On an individual level, challenges with RTW cause feelings of lack of control and increased levels of uncertainty about employment and finances [15]. Since the risk of LC is greater in females, they will likely be disproportionately affected by the illness’s subsequent impacts on loss of employment and income [12,18]. This creates a compounding societal issue as females were already more vulnerable than males in terms of income and employment prior to the pandemic [19]. Further, individuals who intersect multiple vulnerable groups at higher risk for COVID-19 exposure (eg, ethnic monitories, new immigrants, those working in health care settings) often have less access to jobs with modifications or accommodations to promote RTW [20]. Maintaining linkages with the workplace and returning to work as soon as safely possible helps avoid the long-term health and socioeconomic consequences that accompany prolonged unemployment [21].

To optimally tailor rehabilitation programs to LC sequelae and help improve outcomes of RTW programs, more research on LC rehabilitation is needed. This is especially true of rehabilitation programs that specifically aim to promote RTW. It is also important to explore whether certain individuals with LC fare better in rehabilitation than others, as this may identify potentially modifiable lifestyle or broader contextual factors that may facilitate the tailoring of rehabilitation services, thus increasing relevance and potentially improving RTW outcomes in this population.

### Objectives

This study aims to describe the characteristics and outcomes of workers participating in occupational rehabilitation through Workers’ Compensation Board of Alberta’s (WCB-Alberta) Millard Health post-COVID rehabilitation program. We met this aim by (1) describing the characteristics of workers who accessed the program, (2) describing and comparing program admission and discharge data to determine whether there were significant changes in rehabilitation outcomes over the course of the program, and (3) comparing baseline and RTW status at discharge to determine what factors identified through admission data, if any, best predicted RTW status.

The specific research questions (RQs) were as follows:

- RQ1: What are the descriptive characteristics of workers participating in WCB-Alberta’s Millard Health post-COVID rehabilitation program?
- RQ2: Are there significant improvements in outcomes between admission and discharge from the program?
- RQ3: Are worker descriptive characteristics or health status, identifiable upon admission, predictive of RTW status at discharge from the program?

## Methods

### Design

A descriptive cohort study was conducted using data collected by WCB-Alberta for regular program evaluation purposes.

### Ethical Considerations

This research was approved by the University of Alberta’s Health Research Ethics Board (#Pro00113982).

### Population

This study included data from workers participating in WCB-Alberta’s Millard Health post-COVID rehabilitation program. This program was created to help workers with compensation claims due to workplace COVID-19 exposure who developed LC return to regular work duties [22]. The multidisciplinary program consists of occupational, physical, and exercise therapy along with psychology, nursing, and medical interventions, as needed. The program provides psychoeducational approaches for management of LC symptoms, guidance on pacing and energy conservation, and breathing strategies. Some activity or exercise interventions are also prescribed, as tolerated by the workers and in a manner that avoids the postexertional malaise that is common to the LC population. The programs are provided in person, through telerehabilitation (telephone or videoconference), or a combination of the 2, depending on each worker’s individual context. A primary goal of the program is RTW; thus, advice about work activity, exploration of modified duties, and negotiation with employers about appropriate duties are also performed.

The data set included information about all workers who contracted COVID-19 between March 2020 and mid-May 2021. To be included in this study, workers had to be at least 18 years of age and discharged from the aforementioned rehabilitation program. Workers who had not yet been discharged from the program were excluded as their outcomes resulting from program participation were not yet known. All workers had been discharged from the program prior to early January 2022.

### Data Collection Procedures

Anonymized data were extracted from provincial databases managed by WCB-Alberta Health Care Strategy. WCB-Alberta reports are electronic, and data from health care providers are automatically entered into databases. Reports are filed by health care providers at admission to, and discharge from, any WCB-Alberta program. Providers of the post-COVID rehabilitation program report on a variety of demographic, clinical, and occupational variables. Our team has previously conducted several studies using data from WCB-Alberta programs [23-26], and we worked with the same experienced team in Health Care Strategy to retrieve data for this study.

### Sampling

All data points were included in descriptive statistical calculations. This allowed us to obtain a clear picture of the demographics and general outcomes of the post-COVID rehabilitation program. No sample sizes were calculated, as all workers completing the post-COVID rehabilitation program were included (ie, population based).

### Measures

#### Independent Variables

The data set included a variety of descriptive variables, including demographic factors (eg, age, gender), occupational factors (eg, National Occupational Classification code, employment and working status, job attached status, modified work available, work abilities), treatment factors (eg, number and type of services received prior to beginning the post-COVID rehabilitation program, days between date of COVID-19 symptom onset and admission for rehabilitation, program length), and mode of treatment delivery (ie, virtual, in person, or combination). Gender was treated as a categorical variable with 3 options: male, female, and undisclosed.

Independent variables also included patient-reported outcome measures (PROMs) administered at the time of admission to the program. The PROMs included in this study were the Fatigue Severity Scale (FSS) [27], the Post-COVID Functional Scale (PCFS) [28], the 36-item Short Form Health Survey (SF-36) [29], the Pain Disability Index (PDI) [30], the pain Visual Analogue Scale (VAS) [31], the 9-item Patient Health Questionnaire (PHQ-9) [32], the 7-item Generalized Anxiety Disorder Questionnaire (GAD-7) [33], and the *Diagnostic and Statistical Manual for Mental Disorders Fifth Edition* (DSM-5) posttraumatic stress disorder (PTSD) checklist (PCL-5) [34]. Table 1 contains detailed information about each measure. Since the PROMs rely on self-reporting and completion is voluntary, there is typically a high level of missing data on these measures.

**Table 1:** Details about scoring of PROMs^a^ completed by workers in WCB-Alberta’s^b^ Millard Health post-COVID rehabilitation program.

Survey	Measure details
FSS^c^	The FSS contains 9 numerical rating scales, with scores on each scale ranging from 1 (indicating strongly disagree) to 7 (indicating strongly agree) [27]. The 9 scales address the perceived level of disability caused by fatigue as well as how fatigue interferes with physical functioning and activities of daily living [27]. Raw scores are summed into a total score out of 63, with higher scores indicating greater impairment due to fatigue [27].
PCFS^d^	The PCFS is a 1-item question asking “how much the patient is affected in their everyday life by COVID-19” [28]. Scores range from 0 (indicating no functional limitations) to 4 (indicating severe functional limitations) [28].
SF-36^e^	The SF-36 is a 36-item survey that includes domains related to the health-related quality of life specifically in terms of physical functioning, role limitations due to physical health, role limitations due to emotional problems, vitality (ie, energy/fatigue), emotional well-being, social functioning, pain, and general health [29]. Domains are scored, standardized [35,36], and combined into an overall PCS^f^ and an MCS^g^ [29].
PDI^h^	The PDI is a 7-item measure assessing the degree to which pain interferes with family and home responsibilities, recreation, social activity, occupation, sexual behavior, self-care, and activities of daily living [30]. Each item is measured on a scale from 0 (indicating no disability) to 10 (indicating the worst disability) [30]. Raw scores are summed into a total score out of 70, with higher scores indicating greater disability due to pain [30].
Pain VAS^i^	The pain VAS measures a patient’s perceived pain intensity on a scale of 0-100, with 100 indicating the highest level of pain [31].
PHQ-9^j^	The PHQ-9 is a 9-item measure assessing levels of depression [32]. Each item is scored from 0 (indicating not at all) to 4 (indicating nearly every day) [26]. Raw scores are summed into a total score out of 27, with higher scores indicating a higher severity of depression [32].
GAD-7^k^	The GAD-7 is a 7-item measure assessing levels of anxiety [33]. Each item is scored from 0 (indicating not at all) to 3 (indicating nearly every day) [33]. Raw scores are summed into a total score out of 21, with higher scores indicating a higher severity of anxiety [33].
PCL-5^l^	The PCL-5 is a 20-item measure assessing the DSM-5’s^m^ 20 symptoms of PTSD^n^ [34]. Each item is scored from 0 (indicating not at all) to 4 (indicating extremely). Total scores range from 0 to 80, with higher scores indicating a higher likelihood of PTSD [34].

^a^PROM: patient-reported outcome measure.

^b^WCB-Alberta: Workers’ Compensation Board of Alberta.

^c^FSS: Fatigue Severity Scale.

^d^PCFS: Post-COVID Functional Scale.

^e^SF-36: 36-item Short Form Health Survey.

^f^PCS: physical component score.

^g^MCS: mental component score.

^h^PDI: Pain Disability Index.

^i^VAS: Visual Analogue Scale.

^j^PHQ-9: 9-item Patient Health Questionnaire.

^k^GAD-7: 7-item Generalized Anxiety Disorder Questionnaire.

^l^PCL-5: *Diagnostic and Statistical Manual for Mental Disorders Fifth Edition* posttraumatic stress disorder checklist.

^m^DSM-5: *Diagnostic and Statistical Manual for Mental Disorders Fifth Edition.*

^n^PTSD: posttraumatic stress disorder.

#### Dependent Variable

The outcome variable for this study was RTW status at program discharge. RTW status was chosen as the outcome variable of interest because previous research has shown that RTW status is impacted by LC and RTW is a primary goal of the rehabilitation program [14-17]. RTW status was coded as a binary variable, with 1 indicating RTW and 0 indicating “other” (“other” indicated the worker was fit for work [FFW] but had not returned to work or that they were unable to work). We chose to collapse FFW with unable to work due to a low sample size (only 18 cases of FFW) and because those deemed FFW at discharge often have ongoing issues that prevent them from returning to their usual employment. Comparisons were made between the RTW, FFW, and unable-to-work groups on each of the descriptive variables and PROMs. The FFW group was more similar to the unable-to-work group than the RTW group on several of the descriptive variables (ie, occupation, gender, program length, and availability of modified duties). Clinically, the FFW group was also similar to the unable-to-work group on the PDI, FSS, PHQ-9, GAD-7, and PCL-5, further justifying the collapsing of these 2 groups.

### Data Analysis

Data were analyzed using SPSS Statistics version 28 (IBM Corp). To address RQ1, we calculated the mean and SDs of any interval data (eg, worker age) and the frequency of any categorical data (eg, gender or occupation).

To address RQ2, we calculated descriptive statistics for the various PROMs. We calculated the mean and SDs of interval data and the frequency of categorical data. We performed paired-samples *t* tests for each variable collected upon admission to and discharge from the program to determine whether there were any significant improvements in outcomes. Wilcoxon signed-rank tests were performed if the dependent variable was not continuous (ie, the PCFS does not have a total score and therefore is an ordinal variable).

To address RQ3, logistic regression analyses were used to determine which variables (ie, worker demographics, data collected at admission), if any, were predictive of RTW status at discharge. Imputation techniques were used to address the high levels of missing data on the PROMs. We completed univariable logistic regression analyses to examine each potential prognostic factor. Due to the limited sample size, we were unable to build multivariable predictive models. However, we examined the potential confounding effects of age and gender on the significantly predictive variables. Relevant assumptions were tested.

## Results

### Demographics

The data set included 81 workers who had been discharged from WCB-Alberta Millard Health post-COVID program (demographics shown in Table 2). The majority were female (n=52, 64%), had their program delivered virtually (n=79, 98%), and worked in health occupations (n=43, 53%). The mean (SD) age was 48.9 (10.5) years, and the mean (SD) length of time between symptom onset and program admission was 165.2 (73.0) days. Prior to starting the post-COVID program, the workers most frequently visited their doctor (n=64, 79%) or received physiotherapy (n=38, 47%). Although the majority were still employed at program admission (n=77, 95%), only 42 (52%) had modified duties available. A small majority (n=43, 53%) returned to work at the time of program discharge. Of those who returned to work, 40 (93%) returned to modified duties.

**Table 2:** Demographics of workers (N=81) undergoing WCB-Alberta’s^a^ Millard Health post-COVID rehabilitation program.

Variable		Value
Age (years), mean (SD)		48.9 (10.5)
**Gender, n (%)**		
	Male	20 (25)
	Female	52 (64)
	Undisclosed	9 (11)
Duration (average days between symptom onset and admission), mean (SD)		165.2 (73.0)
Program length (work days), mean (SD)		49.9 (12.5)
**Program delivery, n (%)**		
	Virtual	79 (98)
	In person	0 (0)
	Combination	2 (2)
**Occupation category, n (%)**		
	Business, finance, and management	5 (6)
	Health	43 (53)
	Education, law, social, and community government services	10 (12)
	Trades	15 (19)
	Other	8 (10)
**Interpreter required, n (%)**		
	Yes	1 (1)
	No	80 (99)
**Services received prior to admission, n (%)**		
	Physician	63 (79)
	Physiotherapy	38 (47)
	RTW^b^ specialist	27 (33)
	Psychology	26 (32)
	Occupational therapy	19 (24)
	Hospital admission	14 (17)
	Diagnostic testing	19 (24)
	Acupuncture	1 (1)
	Chiropractor	1 (1)
	Injections	1 (1)
	No services prior to admission	8 (10)
**Employed at admission, n (%)**		
	Yes	77 (95)
	No	4 (5)
**Modified duties available at admission, n (%)**		
	Yes	42 (52)
	No	39 (48)
**Work abilities (National Occupational Classification strength level) at admission, n (%)**		
	Limited (lifting required up to 5 kg)	56 (69)
	Light (lifting required up to 10 kg)	8 (10)
	Medium (lifting required up to 20 kg)	3 (4)
	Heavy (lifting required over 20 kg)	4 (5)
	N/A^c^	10 (12)
**Working at admission, n (%)**		
	Yes	10 (12)
	No	71 (88)
**Employed at discharge, n (%)**		
	Yes	76 (94)
	No	5 (6)
**Modified duties available at discharge, n (%)**		
	Yes	50 (62)
	No	31 (38)
**Discharge outcome, n (%)**		
	RTW	43 (53)
	Other	38 (47)
**RTW outcome at program discharge (N=43)**		
	Return to regular work duties	3 (7)
	Return to modified duties	40 (93)
**Work abilities (National Occupational Classification strength level) at discharge**		
	Limited (lifting required up to 5 kg)	41 (51)
	Light (lifting required up to 10 kg)	15 (18)
	Medium (lifting required up to 20 kg)	8 (10)
	Heavy (lifting required over 20 kg)	14 (17)
	N/A	3 (4)

^a^WCB-Alberta: Workers’ Compensation Board of Alberta.

^b^RTW: return-to-work.

^c^N/A: not applicable.

### Patient-Reported Outcome Measures

There were substantial missing data on the PROMs, with 58 (72%) workers not completing at least 1 of the measures at admission or discharge. Only raw SF-36 data were available at discharge, thus preventing calculation of domain scores at admission. However, overall PCSs and MCSs for the SF-36 were logged at admission and discharge.

Table 3 outlines mean (SD) admission and discharge scores on each PROM for those with complete data. Mean (SD) scores on the FSS were quite high at admission (mean 51.3, SD 11.4), indicating moderate-to-severe levels of fatigue in the sample. Pain seemed to cause moderate disruptions in the sample, with a mean (SD) PDI score of 33.3 (15.6) out of 70. Individuals moved from moderate depression (mean 14.1, SD 5.9) to mild depression (mean 10.1, SD 5.3) between admission and discharge, respectively.

Paired-samples *t* tests were run on those with complete matched PROM data (ie, complete data at admission and discharge). Due to the substantial amount of missing PROM data, we included all workers with complete data (the maximum number of matched pairs on any PROM in our sample was 39). Significant changes were noted on several measures (Table 4). There were statistically significant improvements on the pain VAS (mean 11.1, SD 25.6, t_31_=2.5, *P*=.02), the PDI (mean 9.4, SD 12.5, t_32_=4.3, *P*<.001), the FSS (mean 3.9, SD 8.7, t_38_=2.8, *P*=.01), the SF-36 PCS (mean 4.8, SD 8.7, t_38_=–3.5, *P*=.001), the PHQ-9 (mean 3.7, SD 4.0, t_31_=5.2, *P*<.001), and the GAD-7 (mean 1.8, SD 4.4, t_22_=1.8, *P*=.03). There were no significant improvements to the overall MCS measured through the SF-36 or the PCL-5 scores.

The PCFS does not have a total score, so a paired-sample *t* test could not be carried out. Instead, a Wilcoxon signed-rank test was performed (Table 5). Again, due to the substantial amount of missing PROM data, we included only workers with complete matched data (n=38, 47%). There was not a significant difference in PCFS scores between admission and discharge.

We conducted a missing data analysis to determine whether workers with missing data were more or less likely to RTW at discharge. Incomplete data at admission or discharge or both on GAD-7 were significantly associated with RTW (odds ratio [OR] 0.34, 95% CI 0.13-0.87), suggesting that those with incomplete data had a lower likelihood of returning to work.

**Table 3:** Mean scores on PROMs^a^ at the time of admission and discharge from WCB-Alberta’s^b^ Millard Health post-COVID rehabilitation program.

PROMs		Admission	Discharge
**PCFS^c^ (out of 4)**			
	Score, mean (SD)	2.2 (0.8)	2.1 (1.1)
	Missing, n (%)	43 (53)	55 (68)
**Pain VAS^d^ (out of 100)**			
	Score, mean (SD)	48.2 (23.0)	42.0 (25.6)
	Missing, n (%)	25 (31)	39 (48)
**PDI^e^ (out of 70)**			
	Score, mean (SD)	33.3 (15.6)	26.8 (16.2)
	Missing, n (%)	19 (23)	42 (52)
**FSS^f^ (out of 63)**			
	Score, mean (SD)	51.3 (11.4)	48.3 (12.0)
	Missing, n (%)	13 (16)	36 (44)
**SF-36^g^ version 2 (all out of 100), mean (SD)**			
	Physical functioning	N/A^h^	32.9 (11.7)
	Role physical	N/A	35.3 (7.3)
	Role emotional	N/A	42.9 (6.3)
	Bodily pain	N/A	29.7 (5.7)
	Vitality	N/A	33.5 (10.8)
	Social functioning	N/A	29.9 (12.5)
	Mental health	N/A	38.4 (13.3)
	General health perceptions	N/A	32.2 (15.9)
	Overall PCS^i^	28.9 (8.5)	33.4 (9.4)
	Overall MCS^j^	35.2 (11.0)	37.9 (9.0)
**PHQ-9^k^**			
	Score, mean (SD)	14.1 (5.9)	10.1 (5.3)
	Missing, n (%)	39 (48)	43 (53)
**GAD-7^l^**			
	Score, mean (SD)	10.6 (5.0)	8.2 (5.2)
	Missing, n (%)	39 (48)	43 (53)
**PCL-5^m^**			
	Score, mean (SD)	32.4 (15.8)	28.0 (13.2)
	Missing, n (%)	50 (62)	48 (59)

^a^PROM: patient-reported outcome measure.

^b^WCB-Alberta: Workers’ Compensation Board of Alberta.

^c^PCFS: Post-COVID Functional Scale.

^d^VAS: Visual Analogue Scale.

^e^PDI: Pain Disability Index.

^f^FSS: Fatigue Severity Scale.

^g^SF-36: 36-item Short Form Health Survey.

^h^N/A: not applicable.

^i^PCS: physical component score.

^j^MCS: mental component score.

^k^PHQ-9: 9-item Patient Health Questionnaire.

^l^GAD-7: 7-item Generalized Anxiety Disorder Questionnaire.

^m^PCL-5: *Diagnostic and Statistical Manual for Mental Disorders Fifth Edition* posttraumatic stress disorder checklist.

**Table 4:** Mean differences in PROM^a^ scores between admission and discharge from WCB-Alberta's^b^ Millard Health post-COVID rehabilitation program (paired-sample *t* tests).

Variable	Differences, mean (SD)	*t* (*df*)	Two-sided *P* value
Pain VAS^c^ (n=32)	11.1 (25.6)	2.5 (31)	.02
PDI^d^ (n=33)	9.4 (12.5)	4.3 (32)	<.001
FSS^e^ (n=39)	3.9 (8.7)	2.8 (38)	.01
Overall PCS^f^ (n=39)	–4.8 (8.7)	–3.5 (38)	.001
Overall MCS^g^ (n=38)	–0.7 (13.3)	–0.3 (37)	.73
PHQ-9^h^ (n=32)	3.7 (4.0)	5.2 (31)	<.001
GAD-7^i^ (n=32)	1.8 (4.4)	2.3 (31)	.03
PCL-5^j^ (n=23)	5.6 (3.1)	1.8 (22)	.09

^a^PROM: patient-reported outcome measure.

^b^WCB-Alberta: Workers’ Compensation Board of Alberta.

^c^VAS: Visual Analogue Scale.

^d^PDI: Pain Disability Index.

^e^FSS: Fatigue Severity Scale.

^f^PCS: physical component score.

^g^MCS: mental component score.

^h^PHQ-9: 9-item Patient Health Questionnaire.

^i^GAD-7: 7-item Generalized Anxiety Disorder Questionnaire.

^j^PCL-5: *Diagnostic and Statistical Manual for Mental Disorders Fifth Edition* posttraumatic stress disorder checklist.

**Table 5:** Mean differences in PCFS^a^ scores between admission and discharge from WCB-Alberta's^b^ Millard Health post-COVID rehabilitation program (Wilcoxon signed-rank test).

Variable	Admission	Discharge	Median	*Z* value	Two-sided *P* value
Difference	Mean 2.2 (SD 0.8)	Mean 2.1 (SD 1.1)	Admission: 2.0 Discharge: 2.0	–0.6	.53
Missing, n (%)	43 (53)	55 (68)	N/A^c^	N/A	N/A

^a^PCFS: Post-COVID Functional Scale.

^b^WCB-Alberta: Workers’ Compensation Board of Alberta.

^c^N/A: not applicable.

### Predicting Return-to-Work After Rehabilitation

Univariate associations between all potential predictors and the outcome of RTW are shown in Table 6. Three factors were significantly associated with RTW: modified duties available at admission (OR 3.20, 95% CI 1.29-7.95), days between symptom onset and program admission (OR 0.93, 95% CI 0.87-0.998), and the PHQ-9 score at admission (OR 0.87, 95% CI 0.76-0.999). Modified duties available at admission remained a significant predictor of RTW (OR 3.38, 95% CI 1.26-9.10) when controlling for age and gender. Days between symptom onset and program admission also remained a significant predictor of RTW (OR 0.94, 95% CI 0.88-0.999). The PHQ-9 score at admission did not remain significant when controlling for age and gender, suggesting that these demographic variables have a confounding effect. There were no statistically significant or clinically important associations found between any preadmission health care use variable and future RTW status. Imputation with mean (SD), minimum, and maximum values for those with missing data on the PROMs did not result in meaningful changes to the logistic regression analyses. Therefore, we did not present imputed analyses.

**Table 6:** Logistic regression predicting RTW^a^ at time of discharge from WCB-Alberta’s^b^ Millard Health post-COVID rehabilitation program.

Variable		OR^c^ (95% CI)
**Gender**		
	Male	1.00 (N/A^d^)
	Female	1.03 (0.37-2.11)
	Undisclosed	0.41 (0.08-2.91)
Age (years)		0.99 (0.95-1.04)
**Job attached at admission**		
	No	1.00 (N/A)
	Yes	3.60 (0.36-36.17)
**Modified duties available at admission**		
	No	1.00 (N/A)
	Yes	3.20 (1.29-7.95)^e^
Days between symptom onset and admission to program		0.93 (0.87-0.998)^f^
**Work abilities at admission**		
	Heavy	1.00 (N/A)
	Medium	0.17 (0.006-4.52)
	Light	0.33 (0.02-4.74)
	Limited	0.40 (0.04-4.05)
	N/A	0.27 (0.02-3.65)
**Industry**		
	Other	1.00 (N/A)
	Business, finance, and management occupations	6.67 (0.49-91.33)
	Health occupation	2.32 (0.49-10.95)
	Education, law, social, and community government services	2.50 (0.37-16.89)
	Trades	0.83 (0.14-4.99)
**PROMs^g^**		
	PCFS^h^ (n=38), 0-1	1.00 (N/A)
	PCFS (n=38), 2-3	2.57 (0.41-16.12)
	Pain VAS^i^ at admission (n=56)	1.00 (0.98-1.02)
	PDI^j^ at admission (n=62)	0.97 (0.94-1.00)
	FSS^k^ at admission (n=68)	0.96 (0.92-1.01)
	Overall PCS^l^ at admission (n=39)	1.01 (0.96-1.07)
	Overall MCS^m^ at admission (n=38)	1.01 (0.97-1.06)
	PHQ-9^n^ at admission (n=42)	0.87 (0.76-1.00)^e^
	GAD-7^o^ at admission (n=42)	0.88 (0.77-1.02)
	PCL-5^p^ at admission (n=31)	0.97 (0.94-1.03)

^a^RTW: return-to-work.

^b^WCB-Alberta: Workers’ Compensation Board of Alberta.

^c^OR: odds ratio.

^d^N/A: not applicable.

^e^Indicates significance at *P*<.01.

^f^Indicates significance at *P*<.05.

^g^PROM: patient-reported outcome measure.

^h^PCFS: Post-COVID Functional Scale.

^i^VAS: Visual Analogue Scale.

^j^PDI: Pain Disability Index.

^k^FSS: Fatigue Severity Scale.

^l^PCS: physical component score.

^m^MCS: mental component score.

^n^PHQ-9: 9-item Patient Health Questionnaire.

^o^GAD-7: 7-item Generalized Anxiety Disorder Questionnaire.

^p^PCL-5: *Diagnostic and Statistical Manual for Mental Disorders Fifth Edition* posttraumatic stress disorder checklist.

## Discussion

### Principal Findings

In this cohort study of workers with LC participating in WCB-Alberta’s Millard Health post-COVID rehabilitation program, many worker outcomes significantly but modestly improved between admission and discharge. However, several key functional measures did not improve (ie, the PCFS; the overall MCS, measured through the SF-36; and PTSD, measured through the PCL-5). Only a small majority of the sample returned to work (53%), and of these, 93% required modified duties. Those who identified at admission that modified duties were available in their workplace were 3.38 times as likely than those without available modified duties to RTW at program discharge, after controlling for age and gender. Workers with a longer time between symptom onset and program admission also had a lower likelihood of successful RTW.

Our study found that the presence of modified duties in the workplace at admission to LC rehabilitation results in better RTW outcomes. Although we could not find other studies quantifying the relationship between modified duties and RTW with LC, the emerging literature suggests that individuals with LC would likely have greater chances of RTW if they have access to flexible, gradual RTW plans with modified duties. For example, Wong et al [37] completed 2 focus groups (n=8) with rehabilitation counsellors and physicians providing services to individuals with LC and determined that modified work and gradual RTW plans are the most frequently used accommodations to assist individuals with LC with RTW. In a cross-sectional, mixed methods study (N=145) aimed at understanding experiences of workers with LC, Lunt et al [38] found that individuals with LC wanted workplace accommodations that included modified or reduced hours and workload as well as gradual and flexible RTW planning. Support for similar workplace accommodations was echoed in the United Kingdom’s Health and Safety Executive’s report on RTW after LC [39].

The importance of modified duties in LC rehabilitation is consistent with the broader field of occupational rehabilitation and work disability prevention, where modified work duties and RTW coordination are core components of rehabilitation and used to promote RTW [40]. Early intervention is another core principle of occupational rehabilitation [41] and consistent with our finding that more time between initial symptom onset and program admission leads to worse RTW outcomes. However, to meet the clinical case definition of LC (symptoms lasting for at least 3 months after acute infection) [2], individuals with LC are often required to wait at least 3 months to access rehabilitation programs. This waiting period may in turn lead to worse RTW outcomes and therefore warrants further research to determine whether earlier educational or other rehabilitation interventions could improve RTW outcomes in people with lingering symptoms after COVID-19 infection who are not yet diagnosed with LC.

To the best of our knowledge, this is the first study examining the predictors of RTW among workers with LC, likely because of the novelty of the condition. However, previous research has examined RTW in individuals with chronic fatigue syndrome, which has been found to have an overlapping clinical presentation with LC [42]. In a longitudinal study (N=508) exploring sociodemographic, work, and clinical characteristics associated with occupational status among individuals with chronic fatigue syndrome, those who returned to work functioned better (as measured by the SF-36) and were younger [43]. Individuals who reported more fatigue (measured by the Chalder Fatigue Questionnaire) or met the criteria for anxiety and depression (measured by the Hospital Anxiety and Depression Scale) were more likely to have stopped working between baseline and follow-up [43]. These findings suggest that levels of fatigue, age, function, anxiety, and depression may be important variables to consider in future studies analyzing prognostic factors of RTW among individuals living with LC.

### Limitations

The primary limitations of this study are the large amount of missing data on the PROMs and the relatively small sample size. Completion of the PROMs was voluntary for patients in the program, which explains the sizeable amount of missing data. Missing data and a modest sample size limited our ability to build multivariate models and limited conclusions that could be drawn from our results. Having incomplete data on the GAD-7 was significantly associated with worse RTW, which suggests that those with missing data had a lower likelihood of returning to work. There are also likely unmeasured factors that influence both completion of the PROMs and RTW that should be further explored. Results are, however, important for individuals with LC due to the novelty of the condition and uncertainty around optimal rehabilitation approaches.

### Conclusion

Workers undergoing LC rehabilitation reported significant but modest improvements on a variety of PROMs, but only 53% of workers with LC returned to work at the time of program discharge. RTW outcomes would likely improve with increased availability of modified duties and timelier rehabilitation. Additional research is needed, including larger observational cohorts with additional variables as well as randomized controlled trials to evaluate the effectiveness of LC rehabilitation.

## Abbreviations

DSM-5: *Diagnostic and Statistical Manual for Mental Disorders Fifth Edition*
FFW: fit for work
FSS: Fatigue Severity Scale
GAD-7: 7-item Generalized Anxiety Disorder Questionnaire
LC: long COVID
MCS: mental component score
PCFS: Post-COVID Functional Scale
PCL-5: *Diagnostic and Statistical Manual for Mental Disorders Fifth Edition* posttraumatic stress disorder checklist
PCS: physical component score
PDI: Pain Disability Index
PHQ-9: 9-item Patient Health Questionnaire
PROM: patient-reported outcome measure
PTSD: posttraumatic stress disorder
RQ: research question
RTW: return-to-work
SF-36: 36-item Short Form Health Survey
VAS: Visual Analogue Scale
WCB-Alberta: Workers’ Compensation Board of Alberta
